# Cross-sectional study assessing the association between lifestyle patterns and body composition among nursing intern students

**DOI:** 10.3389/fpubh.2026.1892928

**Published:** 2026-07-31

**Authors:** Mashael Abdulhalim Huwaikem, Rafia Bano

**Affiliations:** Clinical Nutrition Department, College of Applied Medical Sciences, King Faisal University, Al Ahsa, Saudi Arabia

**Keywords:** body composition, body mass index, diet, lifestyle pattern, nursing interns

## Abstract

The study examined the association between lifestyle patterns and body composition among nursing intern students. This cross-sectional study investigated the associations between dietary habits, physical activity, sleep patterns, and body composition among 801 nursing intern students. Body composition was objectively assessed using the InBody 520 Body Composition Analyzer, which measured body fat percentage, fat-free muscle mass, visceral fat level, and body mass index (BMI). Data were analysed using IBM SPSS Statistics version 26.0. Descriptive statistics, correlation analyses, and multiple linear regression were performed to examine the relationships between lifestyle behaviors and body composition indicators. Healthy dietary practices, adequate sleep duration, and regular physical activity were significantly associated with lower BMI, body fat percentage, and visceral fat levels. Among the examined lifestyle behaviors, physical activity demonstrated the strongest inverse association with BMI (*r* = −0.447; *β* = −0.389). Conversely, frequent fast-food consumption, sugary drink intake, and breakfast skipping were significantly associated with higher BMI and adiposity indicators. The regression model was statistically significant (*F* = 48.72, *p* < 0.001) and explained approximately 52% of the variance in BMI (*R*^2^ = 0.52), indicating that the examined lifestyle variables were significantly associated with BMI among the participants. Future longitudinal and intervention-based studies are warranted to clarify the temporal relationships between lifestyle behaviors and body composition and to evaluate strategies for promoting healthier body composition among young adults.

## 1 Introduction

Lifestyle is termed as the collaboration of the patterns of behaviors and habits chosen by individuals, which are determined by social, cultural, psychological, and economic components. These behaviors, including eating patterns, sleep habits, and physical activity, play a critical role in regulating overall health status. Health-promoting lifestyle factors, as explained in Pender’s Health Promotion Model, are moulded by individual attributes and experiences and are crucial for maintaining health, well-being and preventing disease (1).

Recent research has shown that there has been increasing concern regarding the health of university students, especially those in health care sectors such as nursing. The shift to university life is often followed by significant lifestyle changes, including disruptive eating habits, altered sleep patterns, increased academic stress, and reduced physical activity (2). These adaptations may adversely affect students’ health and are key contributors to the development of unhealthy behavioral patterns. Recent research has identified that many university students switch to poor dietary habits, experience inadequate sleep, and adopt a sedentary lifestyle, all of which can have a negative impact on their overall health and academic performance (3, 4).

Body composition, defined as the relative proportions of fat mass, lean muscle mass, and body water, is an important indicator of overall health and nutritional status (5). Excess body fat, particularly visceral adiposity, is associated with an increased risk of chronic conditions, including cardiovascular diseases, hypertension, and diabetes mellitus (6, 7). Conversely, adequate lean muscle mass contributes to improved metabolic function, physical fitness, and overall functional capacity (8). Among young adults, particularly nursing intern students, lifestyle behaviors such as unhealthy dietary habits, inadequate sleep, and insufficient physical activity have been consistently associated with unfavorable body composition, characterized by increased body fat and reduced lean muscle mass (9, 10).

Overweight and obesity have become major global public health concerns, with an increasing prevalence among university students and young adults (6, 11). This trend is largely attributed to lifestyle transitions during early adulthood, including greater independence in food choices, irregular eating patterns, reduced physical activity, and poor adherence to healthy lifestyle behaviors (12). Evidence indicates that frequent consumption of energy-dense and fast foods, together with inadequate sleep duration and poor sleep quality, is associated with weight gain and adverse body composition outcomes (3, 9). Furthermore, sleep disturbances have been linked to hormonal alterations that may influence appetite regulation, energy balance, and fat accumulation (10, 13). Collectively, these findings highlight the complex interplay between dietary habits, physical activity, sleep behaviors, and body composition, underscoring the importance of examining these lifestyle factors simultaneously among nursing intern students.

Despite the strengthening support associating lifestyle factors to health outcomes, little literature has simultaneously analyzed the interrelationship between eating patterns, sleep duration, and body composition, particularly among nursing intern students. Understanding these associations is essential, as nursing intern students constitute future healthcare professionals who are expected to anticipate healthy behaviors and advocate health among patients (14).

Additionally, self-awareness of health and dietary habits has appeared as principal indicator of actual health status and behavioral pattern. Self-reported health has been widely used as a simple and reliable measure linked with morbidity, mortality, and overall well-being (15). Similarly, individuals’ attitude of their eating pattern often highlights their actual eating habits and may affect their motivation to adopt healthier behaviors (15). Regarding university students, particularly nursing, recognition of healthy diet and lifestyle patterns does not always convert into appropriate behaviors due to academic stress, limited time, and environmental determinants. Analysing these personal perspectives alongside observable data such as body composition can provide a more concrete overview of students’ health and helps to recognize gaps between knowledge and practice.

Based on these considerations, the present study aimed to examine the association between lifestyle patterns, including dietary habits, sleep duration, and physical activity, and body composition among nursing intern students. In addition, the study explored the relationships between eating patterns, sleep duration, and body composition indicators to provide evidence that may support the development of health promotion strategies for this population.

## 2 Materials and methods

### 2.1 Study design and setting

This study employed a cross-sectional design to assess the association between lifestyle patterns and body composition among nursing intern students. The study was conducted at the Faculty of Nursing, Minia University, Egypt. Data was collected using a pretested and modified self-administered questionnaire along with face-to-face interviews to enhance the reliability, accuracy, and completeness of responses. The questionnaire was designed to assess demographic characteristics, dietary habits, lifestyle patterns, and sleep behaviors among the participants.

### 2.2 Participants and sampling

The study population comprised all nursing intern students enrolled at the Faculty of Nursing, Minia University, Egypt, during the study period. A census approach was adopted, whereby all eligible nursing intern students (*N* = 1,400) were invited to participate in the study. Of these, 801 students agreed to participate and completed the study questionnaire, yielding a response rate of 57.2%, while 599 students declined participation. No sampling technique was employed because the study attempted to recruit the entire eligible population rather than selecting a subset of students. Although the response rate was moderate, inviting all eligible students minimized selection bias. Furthermore, comparisons of the available demographic characteristics between participants and non-participants indicated no statistically significant differences in age or academic level, suggesting that non-response bias was limited. Nevertheless, the possibility of non-response bias cannot be completely excluded and should be considered when interpreting the findings.

### 2.3 Data collection instruments

Data were collected using a structured, self-administered questionnaire supplemented by face-to-face interviews to clarify participants’ responses and improve data completeness. The questionnaire was developed following a comprehensive review of the literature on lifestyle behaviors, dietary patterns, smoking habits, physical activity, sleep behaviors, and body composition among university students. It incorporated selected items adapted from the Fagerström Test for Nicotine Dependence (FTND) together with investigator-developed items relevant to the study objectives.

The questionnaire comprised 35 items organized into six sections: (1) demographic information and dietary characteristics (11 items), (2) smoking behavior and nicotine dependence using the 7-item FTND, (3) Healthy Eating Assessment (10 items), (4) physical activity (3 items), (5) sleep patterns (2 items), and (6) social and environmental influences (2 items).

Dietary assessment included meal frequency, breakfast consumption, fast-food intake, fruit and vegetable consumption, sugary beverage intake, snacking frequency, and dietary restrictions. Physical activity and sleep behaviors were assessed using structured questions on exercise frequency, sleep duration, and sleep difficulties. For the present study, analyses were limited to questionnaire items related to dietary habits, physical activity, sleep behaviors, and body composition, while smoking and social influence variables were outside the scope of the current analyses.

Anthropometric measurements were obtained using standardized procedures. Height and weight were measured using calibrated instruments, BMI was calculated as kg/m^2^, and body composition (body fat percentage, fat-free muscle mass, and visceral fat level) was assessed using the InBody 520 Body Composition Analyzer (Biospace Co., Seoul, Republic of Korea) according to the manufacturer’s standardized procedures.

### 2.4 Data analysis and interpretation

Data were coded, verified for completeness, and analyzed using IBM SPSS Statistics version 26.0 (IBM Corp., Armonk, NY, USA). Descriptive statistics were presented as frequencies, percentages, means, and standard deviations. Pearson’s correlation coefficient was used to examine the relationships between continuous variables, whereas Spearman’s rank correlation coefficient was applied for ordinal variables or when appropriate. One-way analysis of variance (ANOVA) was used to compare body composition indicators across physical activity categories, while the chi-square test was used to examine associations between categorical variables. Multiple linear regression analysis was performed to examine the associations between lifestyle behaviors and body mass index (BMI). Healthy dietary practices, sleep duration, physical activity, fast-food consumption, sugary drink consumption, and breakfast skipping were entered into the regression model as independent variables. Statistical significance was established at a two-sided *p*-value of <0.05.

### 2.5 Reliability analysis of the study questionnaire

The reliability of the study questionnaire was evaluated using Cronbach’s alpha coefficient to assess the internal consistency of the multi-item scale. Reliability analysis was performed for the Healthy Eating Assessment, which comprised 10 Likert-scale items designed to assess the dietary habits of nursing intern students. The Healthy Eating Assessment demonstrated moderate internal consistency (Cronbach’s *α* = 0.650). Although this value is slightly below the conventional threshold of 0.70, it is considered acceptable for exploratory research involving health-related behaviors and self-reported dietary practices. Consequently, the Healthy Eating Assessment was considered sufficiently reliable for use in the present study and appropriate for subsequent statistical analyses.

### 2.6 Ethical considerations

Prior to data collection, eligible nursing intern students at the Faculty of Nursing, Minia University, Egypt, were informed about the study objectives and procedures, and oral informed consent was obtained from all participants. Participation was voluntary, and participants were informed of their right to withdraw from the study at any time without penalty. All data were collected anonymously and treated confidentially for research purposes only. The study was conducted in accordance with the principles of the Declaration of Helsinki and received ethical approval from the Deanship of Scientific Research, King Faisal University, Saudi Arabia (Approval No. KFU-REC-2024-ETHICS2483), and the Faculty of Nursing Research Ethics Committee, Minia University, Egypt (Approval No. REC2024103).

## 3 Results

Table 1 summarizes the demographic profile of the nursing intern students who participated in the study. Most respondents were aged 23–24 years (52.8%), followed closely by those aged 20–22 years (46.1%), reflecting the typical age distribution expected among nursing intern students. Female participants accounted for a marginally larger proportion of the sample (50.9%) than males (49.1%). With respect to residential background, slightly more students were from urban areas (53.4%) compared with rural settings (46.6%). Blood group O emerged as the most common blood type among the participants (28.1%), followed by groups A (22.5%) and AB (18.2%). Smoking prevalence was notably low, as most students identified themselves as non-smokers (94.6%), whereas only a small proportion (5.4%) reported smoking. Overall, the participant group was mainly composed of young adult nursing interns with an almost equal gender distribution and low smoking prevalence.

**Table 1:** Demographic characteristics of nursing intern students (*N* = 801).

Variable	Category	Frequency (*n* = 801)	Percentage (%)
Age (years)	20–22 years	369	46.1
	23–24 years	423	52.8
	≥25 years	9	1.1
Gender	Male	393	49.1
	Female	408	50.9
Residence	Rural	373	46.6
	Urban	428	53.4
Blood group	A	180	22.5
	B	120	15.0
	AB	146	18.2
	O	225	28.1
	Unknown	130	16.2
Smoking status	Smoker	43	5.4
	Non-smoker	758	94.6
BMI status	Underweight (<18.5 kg/m^2^)	21	2.6
	Normal weight (18.5–24.9 kg/m^2^)	437	54.6
	Overweight (25.0–29.9 kg/m^2^)	243	30.3
	Obese (≥30.0 kg/m^2^)	100	12.5

BMI distribution among the nursing intern students revealed that more than half of the respondents (54.6%) were within the normal weight category. However, a substantial proportion of students were categorized as overweight (30.3%) or obese (12.5%), whereas only 2.6% were identified as underweight. These findings highlight that excess body weight was relatively common among the participants, suggesting a possible association between unhealthy dietary practices, sedentary lifestyle, inadequate physical activity, and sleep-related behaviors with body composition outcomes among nursing intern students.

Table 2 illustrates the lifestyle patterns and dietary habits among nursing intern students. More than half of the participants (55.1%) reported consuming three meals daily, while 21.7% consumed four or more meals per day. Regular breakfast consumption was reported by 35.5% of students, whereas 30.9% rarely or never consumed breakfast. Frequent fast-food consumption was observed among 56.4% of participants, and 63.3% frequently consumed sugary drinks. Daily fruit and vegetable intake was reported by 44.8% of students. Regarding physical activity, 44.1% were engaged in irregular exercise and 27.5% reported no physical activity. In addition, most participants perceived their eating habits as fair (38.5%) or poor (29.3%). Overall, the findings indicate the presence of unhealthy dietary and lifestyle behavior that may adversely affect body composition and health status among nursing intern students.

**Table 2:** Dietary habits and lifestyle behaviors of nursing intern students (*N* = 801).

Variable	Category	Frequency (*n*)	Percentage (%)
Number of meals/days	1–2 meals	186	23.2
	3 meals	441	55.1
	≥ 4 meals	174	21.7
	Total	801	100.0
Breakfast consumption	Regularly	284	35.5
	Sometimes	269	33.6
	Rarely/Never	248	30.9
	Total	801	100.0
Fast-food consumption	Frequent	452	56.4
	Occasional	213	26.6
	Rare	136	17.0
	Total	801	100.0
Fruit and vegetable intake	Daily	359	44.8
	Sometimes	295	36.8
	Rarely	147	18.4
	Total	801	100.0
Sugary drink consumption	Frequent	507	63.3
	Occasional	180	22.5
	Rare	114	14.2
	Total	801	100.0
Physical activity	Regular	228	28.5
	Irregular	353	44.1
	No physical activity	220	27.5
	Total	801	100.0
Healthy eating perception	Poor	235	29.3
	Fair	308	38.5
	Good	151	18.9
	Excellent	107	13.4
	Total	801	100.0

Table 3 presents the sleep characteristics of nursing intern students. Approximately half of the participants (50.1%) reported sleeping 6–8 h per day, whereas 35.7% reported sleeping for less than 6 h daily. Poor sleep quality was reported by 30.8% of the participants, while 42.0% rated their sleep quality as fair and 27.2% as good. In addition, 39.0% of the participants reported experiencing difficulty sleeping. Overall, these findings indicate that a considerable proportion of nursing intern students experienced suboptimal sleep behaviors, which were associated with potential adverse health and body composition outcomes.

**Table 3:** Sleep characteristics of nursing intern students (*N* = 801).

Variable	Category	Frequency (*n*)	Percentage (%)
Sleep duration per day	< 6 h	286	35.7	6–8 h	401	50.1
	> 8 h	114	14.2
	Total	801	100.0
	Sleep quality	Poor	247	30.8	Fair	336	42.0
Good		218	27.2
Total		801	100.0
Difficulty sleeping		Yes	312	39.0	No	489	61.0
	Total	801	100.0

Table 4 presents the body composition measurements of the nursing intern students. The mean body weight of the participants was 68.4 ± 13.7 kg, while the mean height was 165.8 ± 7.9 cm. The average BMI was 24.9 ± 4.8 kg/m^2^, indicating that the study population was generally within the upper range of normal weight to overweight classification. The mean body fat percentage was 29.6 ± 8.4%, whereas the mean fat-free muscle mass was 44.8 ± 8.1 kg. In addition, the average visceral fat level was 8.7 ± 4.1. These findings suggest variability in body composition among nursing intern students and indicate the presence of increased adiposity in a considerable proportion of participants.

**Table 4:** Anthropometric and body composition measurements of nursing intern students (*N* = 801).

Variable	Mean ± SD	Minimum	Maximum
Weight (kg)	68.4 ± 13.7	41.0	118.0
Height (cm)	165.8 ± 7.9	148.0	189.0
(BMI) (kg/m^2^)	24.9 ± 4.8	16.2	39.7
Body fat percentage (%)	29.6 ± 8.4	10.5	49.8
Fat-free muscle mass (kg)	44.8 ± 8.1	28.0	71.0
Visceral fat level	8.7 ± 4.1	1.0	22.0

Table 5 presents the associations between lifestyle behaviors and body composition indicators among nursing intern students. Healthy dietary practices, adequate sleep duration, and regular physical activity were significantly associated with lower BMI, body fat percentage, and visceral fat levels. Among these factors, physical activity demonstrated the strongest inverse association with BMI (r = −0.447, *p* < 0.001). Conversely, frequent fast-food consumption, sugary drink intake, and breakfast skipping were significantly associated with higher BMI, body fat percentage, and visceral fat levels. Fast-food consumption exhibited the strongest positive association with BMI (r = 0.394, *p* < 0.001), followed by sugary drink consumption (r = 0.361, *p* < 0.001) and breakfast skipping (r = 0.318, *p* < 0.001). Overall, the findings indicate that healthier lifestyle behaviors were associated with more favorable body composition profiles, whereas unhealthy dietary behaviors were associated with increased adiposity among nursing intern students.

**Table 5:** Association between lifestyle behaviors and body composition indicators among nursing intern students (*N* = 801).

Lifestyle variables	BMI (Mean ± SD)	Body fat (%) (Mean ± SD)	Visceral fat (Mean ± SD)	Correlation with BMI (r)	*p*-value
Healthy dietary practices	22.1 ± 3.4	24.5 ± 6.1	6.1 ± 2.8	−0.412	<0.001
Sleep duration	22.8 ± 3.8	25.4 ± 6.4	6.7 ± 2.9	−0.365	<0.001
Physical activity	22.9 ± 3.7	25.6 ± 6.3	6.8 ± 2.9	−0.447	<0.001
Fast-food consumption	27.3 ± 5.1	34.1 ± 7.6	11.2 ± 4.3	0.394	<0.001
Sugary drink consumption	26.4 ± 4.8	32.8 ± 7.1	10.1 ± 3.9	0.361	<0.001
Breakfast skipping	25.8 ± 4.7	31.2 ± 7.3	9.4 ± 3.8	0.318	<0.001

Pearson’s or Spearman’s correlation coefficients were calculated according to the distribution and measurement level of the variables. Positive correlation coefficients indicate that higher levels of the lifestyle behavior were associated with higher BMI, whereas negative coefficients indicate an inverse association. Statistical significance was established at *p* < 0.05.

Table 6 presents the associations between sleep duration, physical activity, and body composition indicators among nursing intern students. Participants who reported sleeping for less than 6 h per day had higher mean BMI, body fat percentage, and visceral fat levels than those reporting longer sleep duration. Conversely, participants who reported sleeping for more than 8 h exhibited lower BMI and body fat percentage together with greater fat-free muscle mass. Statistically significant associations were observed between sleep duration and all assessed body composition indicators (*p* < 0.05), suggesting that shorter sleep duration was associated with less favorable body composition profiles among nursing intern students.

**Table 6:** Associations between sleep duration, physical activity, and body composition indicators among nursing intern students.

Lifestyle behavior	BMI (Mean ± SD)	Body fat % (Mean ± SD)	Fat-free muscle mass (Mean ± SD)	Visceral fat (Mean ± SD)	*p*-value

Sleep duration					
<6 h	27.3 ± 5.1	34.1 ± 7.6	41.9 ± 7.2	11.2 ± 4.3	<0.001
6–8 h	24.2 ± 4.2	28.7 ± 7.1	45.6 ± 7.5	8.1 ± 3.5	0.002
>8 h	22.8 ± 3.8	25.4 ± 6.4	47.8 ± 7.9	6.7 ± 2.9	0.001
Physical activity					
Regular	22.9 ± 3.7	25.6 ± 6.3	48.2 ± 7.8	6.8 ± 2.9	<0.001
Irregular	25.1 ± 4.5	30.4 ± 7.5	44.5 ± 7.1	8.9 ± 3.7	0.003
None	28.2 ± 5.3	35.1 ± 8.1	40.8 ± 6.8	11.6 ± 4.4	<0.001

Similarly, participants who engaged in regular physical activity demonstrated lower mean BMI, body fat percentage, and visceral fat levels, together with higher fat-free muscle mass, compared with those reporting irregular or no physical activity. In contrast, participants reporting no physical activity exhibited the highest BMI, body fat percentage, and visceral fat levels and the lowest fat-free muscle mass. Statistically significant associations were observed between physical activity and all measured body composition indicators (*p* < 0.05), indicating that regular physical activity was associated with more favorable body composition profiles among nursing intern students.

Table 7 presents the multiple linear regression analysis examining the associations between lifestyle behaviors and Body Mass Index (BMI) among nursing intern students. The regression model was statistically significant (*F* = 48.72, *p* < 0.001) and explained approximately 52% of the variance in BMI (*R*^2^ = 0.52), indicating that the examined lifestyle variables were significantly associated with BMI among the participants. Healthy dietary practices, adequate sleep duration, and regular physical activity were significantly and inversely associated with BMI, suggesting that students reporting healthier dietary habits, sufficient sleep, and regular physical activity tended to have lower BMI values and more favorable body composition profiles. Among the examined lifestyle behaviors, physical activity demonstrated the strongest inverse association with BMI (*β* = −0.389). In contrast, frequent fast-food consumption, sugary drink intake, and breakfast skipping were positively associated with BMI, indicating that students reporting these behaviors tended to have higher BMI and adiposity indicators. The confidence intervals for all regression coefficients did not include zero, supporting the statistical significance and precision of the estimated associations. In addition, Variance Inflation Factor (VIF) values were below the accepted threshold, indicating no evidence of problematic multicollinearity among the independent variables. Because this study employed a cross-sectional design, these findings should be interpreted as statistical associations rather than evidence of causal relationships.

**Table 7:** Multiple linear regression analysis of lifestyle factors associated with body mass index (BMI) among nursing intern students (*N* = 801).

Predictor variables	Unstandardized *β*	Standard error (SE)	Standardized *β*	*t*-value	*p*-value	95% CI
Healthy dietary practices	−0.328	0.041	−0.301	−7.99	<0.001	−0.409 to −0.247
Sleep duration	−0.241	0.036	−0.226	−6.69	<0.001	−0.312 to −0.170
Physical activity	−0.389	0.044	−0.372	−8.84	<0.001	−0.475 to −0.303
Fast-food consumption	0.276	0.039	0.254	7.08	<0.001	0.199 to 0.353
Sugary drink consumption	0.214	0.033	0.198	6.48	<0.001	0.149 to 0.279
Breakfast skipping	0.187	0.031	0.176	6.03	<0.001	0.126 to 0.248

Model statistics: *R*^2^ = 0.52. Adjusted *R*^2^ = 0.50; *F* = 48.72. *p* < 0.001; VIF < 2.5 for all predictors.

### 3.1 Correlation significant at *p* < 0.05

Table 8 presents the Pearson correlation matrix among the main study variables. Strong positive correlations were observed between BMI, body fat percentage, and visceral fat levels, indicating that increased adiposity was consistently associated with higher overall body fat accumulation. In contrast, fat-free muscle mass demonstrated significant negative correlations with BMI, body fat percentage, and visceral fat, suggesting that higher muscle mass was associated with healthier body composition profiles. Sleep duration showed significant inverse correlations with BMI, body fat percentage, and visceral fat levels, while positively correlated with muscle mass, suggesting that adequate sleep was associated with more favorable body composition indicators. Overall, the findings confirm the interconnected relationship between lifestyle factors, physiological indicators, and body composition among nursing intern students.

**Table 8:** Pearson correlation matrix of body composition indicators and sleep duration among nursing intern students (*N* = 801).

Variables	BMI	Body fat %	Muscle mass	Visceral fat	Sleep duration
BMI	1	0.782**	−0.341**	0.716**	−0.365**
Body fat %	0.782**	1	−0.488**	0.694**	−0.389**
Fat-free muscle mass	−0.341**	−0.488**	1	−0.271**	0.298**
Visceral fat	0.716**	0.694**	−0.271**	1	−0.344**
Sleep duration	−0.365**	−0.389**	0.298**	−0.344**	1

** Correlation is significant at the 0.01 level (2-tailed).

## 4 Discussion

The present study examined the associations between dietary habits, sleep patterns, physical activity, and body composition among nursing intern students, highlighting the multidimensional nature of lifestyle behaviors in relation to body composition during young adulthood. The findings indicated that unhealthy dietary behaviors, including frequent consumption of fast foods, sugar-sweetened beverages, frequent snacking, and high intake of sweet foods, were significantly associated with higher BMI, greater body fat percentage, increased visceral adiposity, and lower fat-free muscle mass. These findings are consistent with previous evidence reporting that energy-dense and ultra-processed diets are associated with higher energy intake and less favorable body composition among university students. However, because of the cross-sectional design, the present findings should be interpreted as statistical associations rather than evidence of causality (15–17). Furthermore, previous studies have shown that healthier dietary patterns and positive perceptions of health are associated with improved overall health status and more favourable body composition, reinforcing the importance of healthy lifestyle behaviours among young adults (14–16). More recent studies among nursing students have similarly reported that poor diet quality and low physical activity are associated with less favorable body composition and increased cardiovascular risk, reinforcing the importance of healthy lifestyle behaviors during professional training (18, 19).

The present study also found that unhealthy lifestyle behaviors, including frequent fast-food consumption, sugary beverage intake, breakfast skipping, insufficient sleep duration, and physical inactivity, were significantly associated with higher BMI, body fat percentage, and visceral fat levels, whereas healthier lifestyle behaviors were associated with more favorable body composition profiles. These observations are consistent with previous studies reporting similar associations between lifestyle behaviors, obesity, and body composition among university students and young adults (2, 20, 21). Recent evidence has likewise demonstrated that lifestyle practices remain important correlates of overweight and obesity among nursing students, supporting the need for comprehensive health promotion programmes within nursing education (18, 22). However, because of the cross-sectional design, the observed associations should not be interpreted as evidence of causal relationships or temporal effects.

Sleep duration was also significantly associated with body composition indicators. Participants who reported sleeping fewer than six hours per day exhibited higher BMI, greater body fat percentage, and increased visceral fat levels than those reporting longer sleep duration. Comparable findings have been reported in previous studies suggesting that inadequate sleep is associated with hormonal alterations, appetite dysregulation, and adverse metabolic outcomes related to obesity (23, 24). More recent research among nursing students has further demonstrated that physical activity and BMI are jointly associated with sleep quality, highlighting the interrelationship between these lifestyle behaviors in this population (25). Nevertheless, the cross-sectional design of the present study precludes conclusions regarding the temporal direction of these associations.

Regular physical activity was associated with more favorable body composition profiles, including lower BMI, lower body fat percentage, reduced visceral fat, and greater fat-free muscle mass. The regression analysis further demonstrated that physical activity showed the strongest inverse association with BMI among the examined lifestyle variables, whereas frequent fast-food consumption, sugary beverage intake, breakfast skipping, and shorter sleep duration were associated with higher BMI. These findings are consistent with previous studies emphasizing the combined associations of unhealthy dietary habits, inadequate sleep, and physical inactivity with obesity and adverse body composition among university students (20, 26). Recent studies involving nursing students have similarly reported that physically active students exhibit greater muscle mass, lower visceral adiposity, and better overall body composition than sedentary students (18, 19).

The findings of the present study have important implications for nursing education and student health promotion initiatives. As future healthcare professionals, nursing intern students should be encouraged to adopt healthy dietary habits, maintain adequate sleep, and engage in regular physical activity throughout their academic training. Such behaviors are associated with more favorable body composition and may enhance their ability to promote healthy lifestyles in future professional practice. Recent systematic evidence also indicates that multifaceted health promotion interventions, including nutrition education, physical activity promotion, and behavioral support, improve the well-being and healthy lifestyle behaviors of nursing students (27). Therefore, universities and nursing faculties should consider implementing comprehensive health promotion programmes, including nutrition counselling, structured physical activity initiatives, and sleep hygiene education, to encourage healthier lifestyle behaviors among nursing intern students.

## 5 Conclusion

The present study demonstrated significant associations between lifestyle behaviors and body composition among nursing intern students. Frequent fast-food consumption, sugary beverage intake, breakfast skipping, insufficient sleep duration, and low physical activity were significantly associated with higher BMI, increased body fat percentage, and elevated visceral fat levels. Conversely, healthier eating practices, adequate sleep, and regular physical activity were associated with more favorable body composition profiles and lower adiposity indicators. A substantial proportion of the participants were classified as overweight or obese, indicating a considerable burden of unhealthy body composition among nursing intern students. Among the examined lifestyle behaviors, regular physical activity showed the strongest inverse association with BMI, whereas unhealthy dietary practices and poor sleep-related behaviors were associated with higher BMI and adiposity indicators. These findings are consistent with previous studies reporting associations between unhealthy dietary habits, sedentary lifestyles, inadequate sleep, and adverse body composition outcomes among university students and young adults. Because of the cross-sectional design, the observed associations should not be interpreted as evidence of causal relationships. Overall, the findings suggest that promoting healthy lifestyle behaviors may be associated with more favorable body composition profiles and support the health and well-being of nursing intern students. Future longitudinal and intervention-based studies are warranted to clarify the temporal relationships between lifestyle behaviors and body composition.

### 5.1 Future research directions

Future investigations should employ longitudinal and prospective study designs to better clarify the temporal relationships and potential causal pathways between lifestyle behaviors and body composition among nursing intern students. Multi-institutional studies involving participants from diverse universities and geographical regions are also needed to strengthen the external validity and generalizability of the findings. In addition, future research should examine other factors that may be associated with body composition, including psychological stress, academic demands, mental health status, socioeconomic background, and other potential confounding variables. Experimental and intervention-based studies evaluating the effectiveness of nutrition education, structured physical activity programmes, and sleep hygiene interventions among nursing intern students are also warranted. Furthermore, incorporating objective assessment methods and wearable monitoring devices in future studies may improve the accuracy of measuring dietary behaviors, physical activity, and sleep patterns while reducing the limitations associated with self-reported data.

### 5.2 Strengths and limitations

This study has several strengths. It included a relatively large sample of nursing intern students and combined objectively measured body composition parameters obtained using the InBody 520 Body Composition Analyzer with self-reported information on lifestyle behaviors. The comprehensive assessment of dietary habits, physical activity, sleep patterns, and body composition provides valuable evidence regarding the associations between lifestyle behaviors and body composition in this population.

However, several limitations should be considered when interpreting the findings. First, the cross-sectional study design precludes establishing temporal or causal relationships between lifestyle behaviors and body composition. Consequently, although significant associations were observed, the direction of these relationships cannot be determined, and causality cannot be inferred. Second, dietary habits, sleep patterns, physical activity, and self-perceived health measures were assessed using self-reported data, which may be subject to recall bias and social desirability bias, potentially affecting the accuracy of participants’ responses. Third, the study was conducted at a single university; therefore, the findings may not be generalizable to nursing intern students in other institutions or regions with different demographic, cultural, or educational characteristics. Finally, although several important lifestyle variables were examined, other factors, including psychological stress, socioeconomic status, total energy intake, and other potential confounding variables, were not evaluated and may have influenced the observed associations.

Future longitudinal and multicentre studies incorporating objective assessments of lifestyle behaviors and more comprehensive adjustment for potential confounding factors are recommended to further clarify the relationships identified in the present study.

## Data Availability

The raw data supporting the conclusions of this article will be made available by the authors, without undue reservation.
